# The impact of maternal gestational diabetes mellitus on cardiac structural and functional parameters in infants

**DOI:** 10.3389/fendo.2026.1701975

**Published:** 2026-03-05

**Authors:** Yuhong Deng, Zixiang Wang, Shuangping Guo, Yong Guo, Jie-Ling Wu

**Affiliations:** 1 Department of Children’s Health Care, Guangdong Women and Children Hospital, Guangzhou, China; 2 Department of Adult Surgery, Guangdong Women and Children Hospital, Guangzhou, China; 3 Department of Ultrasonography, Guangdong Women and Children Hospital, Guangzhou, China; 4 Department of Health Care, Guangdong Women and Children Hospital, Guangzhou, China

**Keywords:** cardiac structural and functional parameters, gestational diabetes mellitus (GDM), infant, pregnancy, sex-specific difference

## Abstract

**Objective:**

This study aimed to retrospectively analyze changes in cardiac structural and functional parameters in infants born to mothers with nonpharmacologically managed gestational diabetes mellitus (GDM) to investigate potential cardiovascular developmental risks in GDM offspring beyond overt birth defects and to examine their age and sex specificity.

**Methods:**

This retrospective cohort study included infants aged 1–12 months who underwent routine pediatric health examinations, including cardiac ultrasound screening, at Guangdong Women and Children Hospital from January 2018 to December 2023. Participants were assigned to a GDM group or a non-GDM control group based on maternal diagnosis. Echocardiography measured cardiac parameters. Multivariable linear and logistic regression models assessed the association between GDM exposure and cardiac parameters, with stratification by infant age (< 6 months vs. 6–12 months) and sex.

**Results:**

A total of 11,782 mother–infant pairs were enrolled (1,734 in the GDM group; 10,048 in the non-GDM group). After adjusting for confounders, GDM exposure was significantly associated with larger cardiac dimensions, including increased right atrial dimension (RAD) (adjusted mean difference [aMD] = 0.22, 95% confidence interval [95% CI]: 0.09–0.36, *p* = 0.001), right ventricular outflow tract dimension (aMD = 0.19, 95% CI: 0.08–0.30, *p* = 0.001), left atrial dimension (LAD) (aMD = 0.17, 95% CI: 0.03–0.32, *p* = 0.020), left ventricular end-diastolic dimension (aMD = 0.29, 95% CI: 0.11–0.46, *p* = 0.002), and left ventricular end-systolic dimension (aMD = 0.17, 95% CI: 0.05–0.29, *p* = 0.006). Age-stratified analysis revealed that in infants < 6 months, GDM was associated with increased RAD (aMD = 0.13, 95% CI: 0.01–0.26, *p* = 0.040) and LAD (aMD = 0.19, 95% CI: 0.04–0.33, *p* = 0.013). Conversely, in infants aged 6–12 months, GDM was associated with smaller right ventricular dimension (aMD = − 0.37, 95% CI: − 0.72 to − 0.01, *p* = 0.043) and LAD (aMD = − 0.48, 95% CI: − 0.79 to − 0.17, *p* = 0.002). Sex-stratified analysis showed that the associations between GDM and enlarged cardiac structures were significant only in male infants. Furthermore, GDM significantly increased the odds of extremely high RAD (≥ 95th percentile) in male infants (OR = 1.30, 95% CI: 1.02–1.65). No significant association was found between GDM and left ventricular ejection fraction in any model.

**Conclusions:**

Maternal GDM induces subtle, sex-specific cardiac structural changes in infants, particularly in male infants. Although not clinically overt, these alterations may indicate long-term cardiovascular risk, highlighting the need for longitudinal follow-up and research to clarify their predictive value for future cardiovascular disease.

## Introduction

Since 2011, China has adopted the International Association of Diabetes and Pregnancy Study Groups (IADPSG) criteria as the professional standard for diagnosing gestational diabetes mellitus (GDM) (1). The implementation of universal screening for pregnant women at 24–28 weeks of gestation, using the 75-g oral glucose tolerance test (OGTT) in a “one-step” approach, has significantly enhanced both the detection rate and the standardization of GDM management (2, 3). A recent systematic review and meta-analysis (1990–2024) calculated the pooled GDM prevalence in China, based on the IADPSG criteria, as 15.6% (95% confidence interval [95% CI]: 14.9%–16.2%) (4). Over the past decade, with increased awareness of perinatal healthcare and optimized glycemic management strategies, the majority of GDM patients have achieved effective blood glucose control through medical nutrition therapy, physical activity, and insulin treatment (5–7). Consequently, the incidence of severe adverse pregnancy outcomes traditionally associated with GDM, such as macrosomia and overt congenital malformations, has declined (8).

However, even among GDM mothers with well-controlled blood glucose, the potential effects of the hyperglycemic intrauterine environment on fetal development may not be entirely mitigated. Increasing evidence suggests that *in utero* exposure to hyperglycemia can induce subtle yet persistent alterations in offspring organ systems, particularly the cardiovascular system, through mechanisms such as epigenetic programming and multiple metabolic pathways (9, 10). These effects may not manifest as significant birth defects but rather as subclinical changes in cardiac structure and function (11). Recent advancements in the pathophysiological understanding of GDM suggest that metabolic dysregulation in early pregnancy can adversely affect early placental development, thereby influencing both maternal metabolism and fetal growth (12). Fetal hyperinsulinemia can impact the development of multiple fetal tissues, resulting in both short- and long-term consequences. Although glycemic management throughout gestation can prevent some, but not all, pregnancy complications in GDM patients, recent studies have shown that offspring of GDM mothers may still exhibit minor cardiac abnormalities, such as myocardial hypertrophy and diastolic dysfunction (13–15). The persistence of these changes during infancy remains unclear. At present, large-sample, long-term follow-up studies on cardiac development in the offspring of GDM mothers in China are still lacking. Most existing studies focus on the gestational or neonatal period (16). Infancy (1–12 months of age) represents a critical window for the heart’s transition from the fetal to the mature circulatory pattern, as well as a key period for cardiac remodeling and functional adaptation (17). Investigating potential alterations in cardiac structure and function in offspring of GDM mothers during this stage is crucial for a deeper understanding of the long-term impact of GDM and for refining early cardiovascular health surveillance strategies for this population.

Based on a large sample from the Chinese population in Guangdong Province, this study aimed to retrospectively analyze changes in cardiac structural and functional parameters in infants born to mothers with nonpharmacologically managed GDM. The goal was to elucidate subtle cardiovascular developmental risks in GDM offspring that extend beyond overt birth defects, providing new scientific evidence to inform precise health management and long-term risk prevention for these children.

## Methods

### Study population

Between January 2018 and December 2023, infants aged 1–12 months who received routine pediatric health examinations (including cardiac ultrasound screening) at Guangdong Women and Children Hospital were included in this retrospective cohort study. Maternal medical records were retrospectively reviewed for those who received antenatal care and delivered at the same institution. Data collection involved extracting echocardiographic data for infants aged 1–12 months, maternal information from electronic medical records—including results of the 75-g OGTT performed at 24–28 weeks of gestation—as well as delivery details. The integrity and validity of all collected data were rigorously assessed. The exclusion criteria were as follows: severe neonatal congenital malformations; maternal pregestational diabetes (type 1 or 2); third-trimester Hemoglobin A1c (HbA1c) > 6% or use of insulin therapy during pregnancy; severe chronic maternal diseases (e.g., hypertension, hepatic disease, or renal disease); and incomplete key data (i.e., missing OGTT results, delivery information, or infant cardiac ultrasound data). A flow diagram detailing the participant selection process is shown in **Supplementary Figure 1**.

### Ethical approval

The research protocol underwent review and received full approval from the Institutional Review Board at Guangdong Women and Children Hospital (Approval ID: 202301234). As the investigation relied solely on the analysis of anonymized clinical information sourced from the institution’s existing health records, the requirement for obtaining patient-informed consent was formally waived by the committee. This exemption aligns with established national guidelines governing the use of deidentified data in medical research.

### GDM assessment

Screening for GDM was conducted at 24–28 weeks of gestation using a 75-g OGTT. In accordance with established guidelines (18), a diagnosis of GDM was confirmed if at least one plasma glucose value met or surpassed the following thresholds: fasting ≥ 5.1 mmol/L, 1-h postchallenge ≥ 10.0 mmol/L, or 2-h postchallenge ≥ 8.5 mmol/L. Maternal glycemic status during late pregnancy was assessed by collecting HbA1c levels in the third trimester.

### Cardiac structural and functional parameters

All echocardiographic examinations were conducted by experienced technicians using a Philips IE ELITE ultrasound system, following a well-established standardized institutional protocol. The study incorporated a comprehensive set of cardiac structural and functional parameters. Structural measurements included right atrial diameter (RAD), right ventricular diameter (RVD), right ventricular outflow tract diameter (RVOTD), left atrial diameter (LAD), left ventricular end-diastolic diameter (LVDd), and left ventricular end-systolic diameter (LVDs). Hemodynamic parameters were assessed through Doppler flow velocities, including pulmonary valve (PV) flow velocity, aortic valve (AV) flow velocity, tricuspid valve (TV) flow velocity, and mitral valve E-wave (MVE) velocity. Left ventricular systolic function was evaluated using the left ventricular ejection fraction (LVEF).

### Statistical analysis

All quantitative data, such as maternal age, gestational age at delivery, birth weight and length, and echocardiographic parameters, were initially assessed for normality using the Shapiro–Wilk test. Normally distributed variables were expressed as mean ± standard deviation (SD), and comparisons between groups were conducted using independent samples *t*-tests. Categorical variables, such as maternal education level, parity, and neonatal sex, were reported as numbers and percentages (*n*; %) and compared using the Chi-square (*χ*²) test. A multiple linear regression model was applied to evaluate the association between maternal GDM exposure and infant cardiac structural and functional parameters. The results are presented as adjusted mean differences (aMD) with 95% CIs. The model was adjusted for the following covariates: maternal age, education, pre-pregnancy body mass index (BMI), parity, mode of delivery, HbA1c, gestational age at delivery, birth weight, birth length, infant age, and sex. Furthermore, using the distribution of each echocardiographic parameter from the control group (infants born to mothers without GDM) as the normative reference, extreme values were defined as those at or below the 5th (≤ 5th) percentile or at or above the 95th (≥ 95th) percentile. A multiple logistic regression analysis was conducted to assess the odds of extreme cardiac parameter values in offspring of mothers with GDM compared with those of non-GDM mothers. Results are reported as odds ratios (ORs) with 95% CIs. The covariates included in this model were identical to those used in the linear regression analysis. Subgroup analyses were also performed, stratified by infant age (< 6 and 6–12 months) and sex, to further explore the association between GDM exposure and infant cardiac parameters. All statistical analyses were conducted using RStudio (version 2022.07.0) and SPSS (version 26.0). All hypothesis tests were two-sided, and a *p*-value of less than 0.05 was considered statistically significant. The Benjamini–Hochberg procedure was used to control the false discovery rate (FDR) for multiple comparisons, with significance for this correction set at an FDR-adjusted *p*-value (*q*-value) < 0.05.

## Results

A total of 11,782 mother–infant pairs were enrolled in this study, comprising 1,734 in the GDM group and 10,048 in the non-GDM group (**Table 1**). Significant differences were observed between the two groups across several baseline characteristics (*p* < 0.05). Specifically, mothers in the GDM group were older (31.49 years ± 4.49 years vs. 29.52 years ± 4.23 years), had a higher pre-pregnancy BMI (21.87 kg/m² ± 3.18 kg/m² vs. 20.63 kg/m² ± 2.77 kg/m²), and had higher rates of multiparity (45.6% vs. 38.0%) and cesarean delivery (40.3% vs. 34.2%) compared with the non-GDM group. Furthermore, infants born to mothers with GDM had a lower mean gestational age at delivery (39.00 weeks ± 1.49 weeks vs. 39.29 weeks ± 1.38 weeks) and a higher incidence of preterm birth (5.9% vs. 3.9%). No significant differences were found between the groups in terms of maternal education, neonatal birth weight, or sex distribution (*p* > 0.05).

**Table 1:** Characteristics of the study sample grouped by women with and without GDM.

Characteristics	All (*n* = 11,782)	Non-GDM (*n* = 10,048)	GDM (*n* = 1,734)	*p*-value
Maternal age (years)	29.81 ± 4.32	29.52 ± 4.23	31.49 ± 4.49	< 0.001
Maternal education				
Junior high school or below	1,196 (10.3)	1,021 (10.3)	175 (10.2)	0.922
High school	2,283 (19.6)	1,953 (19.7)	330 (19.3)	
University and above	8,156 (70.1)	6,950 (70.0)	1,206 (70.5)	
Pre-pregnancy BMI (kg/m^2^)	20.81 ± 2.87	20.63 ± 2.77	21.87 ± 3.18	< 0.001
Parity				
Nulliparous	7,178 (60.9)	6,234 (62.0)	944 (54.4)	< 0.001
Multiparous	4,604 (39.1)	3,814 (38.0)	790 (45.6)	
Delivery mode				
Vaginal delivery	7,643 (64.9)	6,607 (65.8)	1,036 (59.7)	< 0.001
Cesarean section	4,139 (35.1)	3,441 (34.2)	698 (40.3)	
OGTT-0h (mmol/L)	4.37 ± 0.33	4.32 ± 0.28	4.64 ± 0.46	< 0.001
OGTT-1h (mmol/L)	7.70 ± 1.62	7.33 ± 1.34	9.92 ± 1.33	< 0.001
OGTT-2h (mmol/L)	6.70 ± 1.36	6.36 ± 1.05	8.71 ± 1.29	< 0.001
HbA1c (%)	4.92 ± 0.34	4.89 ± 0.33	5.07 ± 0.34	< 0.001
Gestational age at birth (weeks)	39.24 ± 1.4	39.29 ± 1.38	39.00 ± 1.49	< 0.001
Birth weight (kg)	3.18 ± 0.43	3.19 ± 0.42	3.18 ± 0.47	0.345
Birth length (cm)	49.43 ± 1.93	49.45 ± 1.91	49.31 ± 2.02	0.005
Preterm birth	490 (4.2)	387 (3.9)	103 (5.9)	< 0.001
Neonatal sex				
Male	5,628 (47.8)	4,826 (48.0)	802 (46.3)	0.171
Female	6,154 (52.2)	5,222 (52.0)	932 (53.7)	

**Figure 1:**
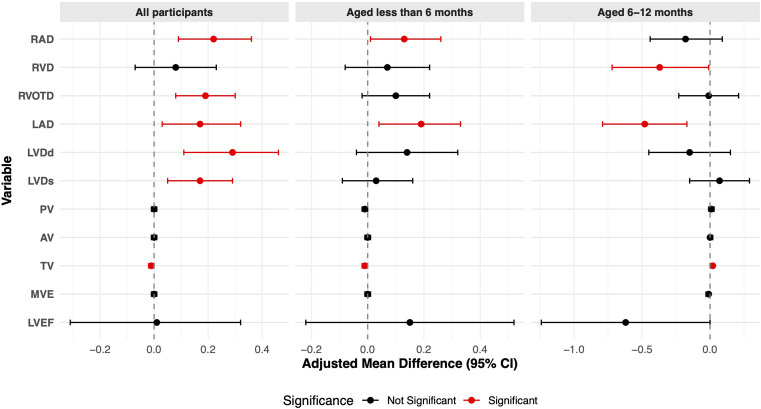
Adjusted mean differences comparing cardiac structure and function parameters in infants exposed to maternal GDM with those in infants not exposed to maternal GDM.

In the overall cohort, infants in the GDM group had significantly larger dimensions for several left- and right-heart structures compared to the non-GDM group (**Table 2**). No statistically significant differences were observed between the groups for any valve flow velocities or for LVEF. After multivariate adjustment, exposure to GDM was significantly associated with alterations in several infant cardiac structural parameters, and this association demonstrated notable age- and sex-specific patterns (**Figure 1**; **Supplementary Table 1**). In the overall cohort analysis, GDM exposure remained associated with significantly larger cardiac dimensions. These included RAD (aMD = 0.22, 95% CI: 0.09–0.36, *p* = 0.001), RVOTD (aMD = 0.19, 95% CI: 0.08–0.30, *p* = 0.001), LAD (aMD = 0.17, 95% CI: 0.03–0.32, *p* = 0.020), LVDd (aMD = 0.29, 95% CI: 0.11–0.46, *p* = 0.002), and LVDs (aMD = 0.17, 95% CI: 0.05–0.29, *p* = 0.006). Additionally, GDM exposure was associated with a significantly lower TV flow velocity (aMD = − 0.01, 95% CI: − 0.02–0, *p* = 0.019). The age-stratified analysis revealed distinct associations. Among infants younger than 6 months, GDM exposure was significantly and positively associated with RAD (aMD = 0.13, 95% CI: 0.01–0.26, *p* = 0.040) and LAD (aMD = 0.19, 95% CI: 0.04–0.33, *p* = 0.013). In this subgroup, TV flow velocity also remained significantly lower (aMD = − 0.01, 95% CI: − 0.02–0, *p* = 0.005). Conversely, among infants aged 6–12 months, GDM was associated with smaller cardiac dimensions, including a significantly smaller RVD (aMD = − 0.37, 95% CI: − 0.72 to − 0.01, *p* = 0.043) and LAD (aMD = − 0.48, 95% CI: − 0.79 to − 0.17, *p* = 0.002). In this older infant group, TV flow velocity was significantly higher compared to the non-GDM group (aMD = 0.02, 95% CI: 0–0.03, *p* = 0.027).

**Table 2:** Comparison of cardiac structure and function parameters between infants of mothers with and without GDM.

Variable	Non-GDM	GDM	*p*-value
All participants (aged 1–12 months)			
RAD (mm)	16.21 ± 2.37	16.40 ± 2.41	0.002
RVD (mm)	12.86 ± 2.67	12.98 ± 2.64	0.070
RVOTD (mm)	11.58 ± 2.02	11.70 ± 2.06	0.023
LAD (mm)	13.95 ± 2.57	14.10 ± 2.55	0.021
LVDd (mm)	21.46 ± 3.16	21.75 ± 3.18	< 0.001
LVDs (mm)	13.58 ± 2.15	13.77 ± 2.21	0.001
PV (m/s)	1.01 ± 0.17	1.01 ± 0.17	0.915
AV (m/s)	1.02 ± 0.14	1.02 ± 0.14	0.610
TV (m/s)	0.71 ± 0.14	0.70 ± 0.14	0.076
MVE (m/s)	0.90 ± 0.15	0.90 ± 0.15	0.889
LVEF (%)	69.44 ± 5.69	69.48 ± 5.58	0.749
Aged less than 6 months (*n* = 9,228)			
RAD (mm)	15.61 ± 1.98	15.7 ± 2.03	0.124
RVD (mm)	12.42 ± 2.34	12.55 ± 2.34	0.061
RVOTD (mm)	11.15 ± 1.80	11.20 ± 1.85	0.384
LAD (mm)	13.38 ± 2.23	13.52 ± 2.23	0.039
LVDd (mm)	20.66 ± 2.77	20.84 ± 2.78	0.025
LVDs (mm)	13.12 ± 1.95	13.20 ± 1.96	0.166
PV (m/s)	1.02 ± 0.17	1.02 ± 0.17	0.974
AV (m/s)	1.01 ± 0.14	1.01 ± 0.14	0.964
TV (m/s)	0.72 ± 0.14	0.71 ± 0.15	0.059
MVE (m/s)	0.89 ± 0.15	0.89 ± 0.15	0.887
LVEF (%)	69.23 ± 5.71	69.38 ± 5.67	0.375
Aged 6–12 months (*n* = 2,554)			
RAD (mm)	18.48 ± 2.35	18.34 ± 2.33	0.254
RVD (mm)	14.51 ± 3.14	14.18 ± 3.06	0.039
RVOTD (mm)	13.19 ± 1.97	13.08 ± 2.00	0.283
LAD (mm)	16.1 ± 2.67	15.73 ± 2.69	0.007
LVDd (mm)	24.5 ± 2.67	24.28 ± 2.86	0.110
LVDs (mm)	15.30 ± 2.00	15.35 ± 2.12	0.685
PV (m/s)	0.99 ± 0.16	1.00 ± 0.17	0.403
AV (m/s)	1.03 ± 0.12	1.04 ± 0.13	0.577
TV (m/s)	0.66 ± 0.11	0.67 ± 0.12	0.069
MVE (m/s)	0.95 ± 0.14	0.94 ± 0.13	0.061
LVEF (%)	70.22 ± 5.52	69.77 ± 5.32	0.108

**Figure 2:**
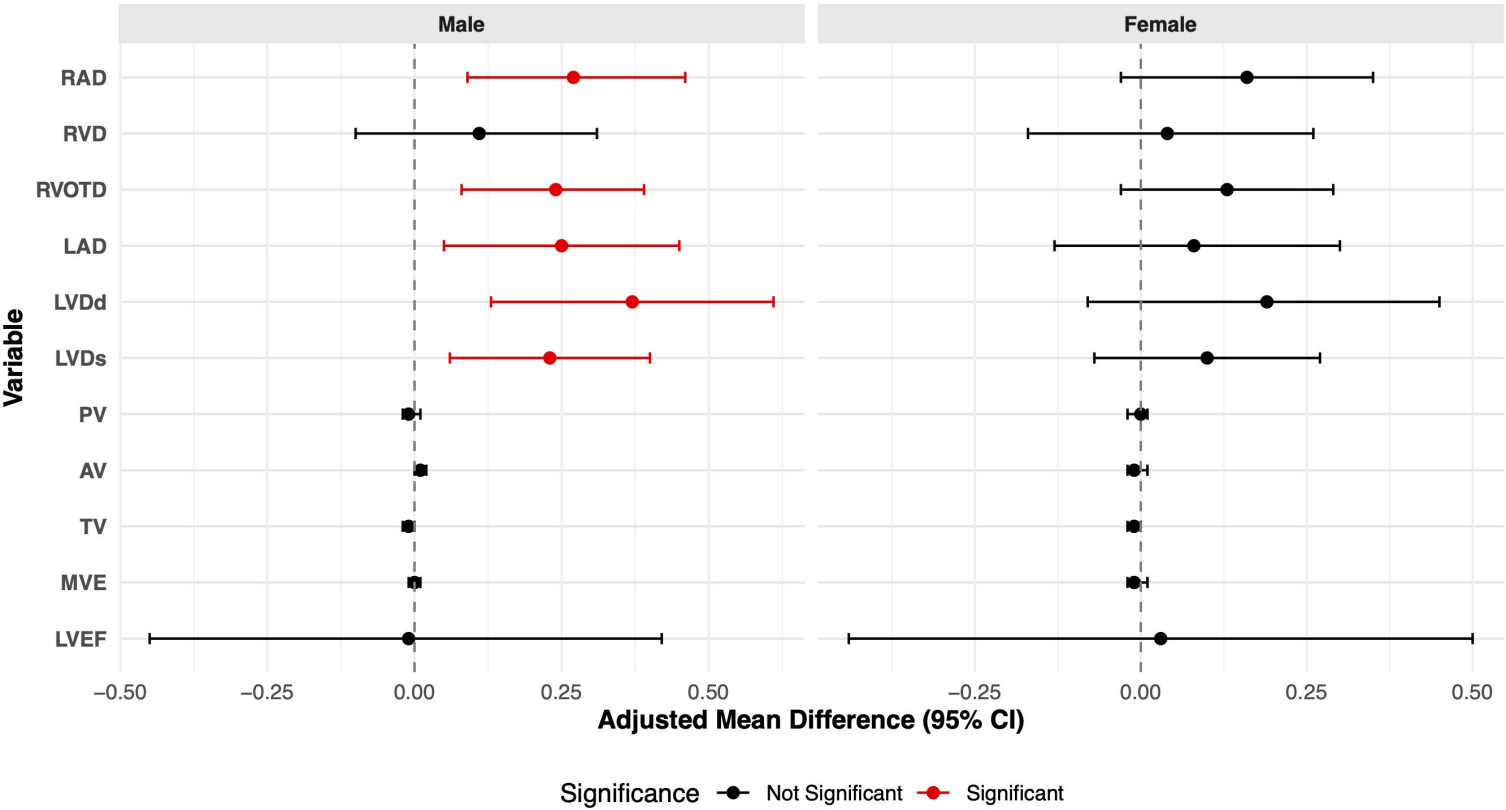
Stratified analysis by sex of adjusted mean differences in cardiac structure and function parameters in infants exposed to maternal GDM compared with those not exposed to maternal GDM.

Stratification by sex indicated that male infants were more susceptible to the effects of GDM exposure on cardiac structure (**Figure 2**; **Supplementary Table 2**). GDM was significantly associated with larger RAD, RVOTD, LAD, LVDd, and LVDs in male infants (all *p* < 0.05). In contrast, none of these associations were statistically significant in female infants. LVEF was not significantly associated with GDM exposure in any of model.

Multivariate-adjusted logistic regression analysis revealed that exposure to GDM was significantly associated with the odds of extreme values for certain infant cardiac parameters, with age- and sex-specific patterns observed (**Table 3**). In the overall population, infants in the GDM group had significantly higher odds of having a RAD at or above the 95th percentile compared with the non-GDM group (OR = 1.29, 95% CI: 1.07–1.56, *p* = 0.008). The odds of having RVOTD ≥ 95th percentile were also significantly increased (OR = 1.24, 95% CI: 1.01–1.51, *p* = 0.036). Furthermore, the GDM group had higher odds of TV flow velocity measurements at or below the 5th percentile (OR = 1.32, 95% CI: 1.04–1.67, *p* = 0.020). Age-stratified analysis indicated that the increased odds of low TV flow velocity were particularly pronounced in infants younger than 6 months (OR = 1.49, 95% CI: 1.13–1.97, *p* = 0.005). In contrast, among infants aged 6–12 months, the odds of having a LAD ≥ 95th percentile were significantly lower (OR = 0.77, 95% CI: 0.59–1.00, *p* = 0.050).

**Table 3:** Odds ratios for extreme values of cardiac structure and function parameters (values ≤ 5th or ≥ 95th percentile) comparing infants born to mothers with GDM and infants born to mothers without GDM.

Variable	All participants		Aged less than 6 months		Aged 6–12 months	
	ORs (95% CI)^a^	*p*-value	ORs (95% CI)^b^	*p*-value	ORs (95% CI)^b^	*p*-value
RAD (mm)						
≤ 5th percentile	0.85 (0.71, 1.03)	0.096	0.87 (0.72, 1.06)	0.162	1.99 (0.78, 5.12)	0.152
≥ 95th percentile	1.29 (1.07, 1.56)	0.008	1.45 (0.99, 2.11)	0.056	1.00 (0.78, 1.28)	0.986
RVD (mm)						
≤ 5th percentile	0.88 (0.70, 1.10)	0.264	0.87 (0.68, 1.12)	0.276	1.24 (0.65, 2.38)	0.510
≥ 95th percentile	1.10 (0.87, 1.39)	0.443	1.32 (0.90, 1.95)	0.159	0.79 (0.58, 1.08)	0.137
RVOTD (mm)						
≤ 5th percentile	0.89 (0.75, 1.05)	0.149	0.90 (0.76, 1.07)	0.242	2.15 (1.00, 4.63)	0.051
≥ 95th percentile	1.24 (1.01, 1.51)	0.036	1.18 (0.85, 1.64)	0.316	1.03 (0.79, 1.35)	0.836
LAD (mm)						
≤ 5th percentile	0.86 (0.69, 1.08)	0.191	0.88 (0.69, 1.11)	0.277	1.93 (0.73, 5.11)	0.183
≥ 95th percentile	1.00 (0.82, 1.22)	0.973	0.98 (0.69, 1.39)	0.922	0.77 (0.59, 1.00)	0.050
LVDd (mm)						
≤ 5th percentile	0.87 (0.70, 1.08)	0.213	0.91 (0.72, 1.13)	0.390	2.32 (0.54, 9.99)	0.257
≥ 95th percentile	0.97 (0.77, 1.23)	0.828	0.69 (0.42, 1.12)	0.132	0.87 (0.65, 1.16)	0.335
LVDs (mm)						
≤ 5th percentile	0.86 (0.72, 1.01)	0.071	0.89 (0.75, 1.07)	0.209	1.01 (0.43, 2.39)	0.980
≥ 95th percentile	1.16 (0.95, 1.41)	0.142	0.94 (0.68, 1.30)	0.707	1.07 (0.82, 1.40)	0.604
PV (m/s)						
≤ 5th percentile	1.15 (0.91, 1.45)	0.257	1.21 (0.92, 1.60)	0.178	1.00 (0.64, 1.57)	0.995
≥ 95th percentile	1.04 (0.81, 1.32)	0.786	1.05 (0.80, 1.39)	0.725	1.00 (0.58, 1.73)	0.988
AV (m/s)						
≤ 5th percentile	0.96 (0.74, 1.23)	0.725	0.96 (0.73, 1.27)	0.796	1.22 (0.63, 2.36)	0.555
≥ 95th percentile	0.97 (0.76, 1.25)	0.818	0.89 (0.67, 1.19)	0.442	1.39 (0.83, 2.31)	0.212
TV (m/s)						
≤ 5th percentile	1.32 (1.04, 1.67)	0.020	1.49 (1.13, 1.97)	0.005	0.92 (0.59, 1.43)	0.705
≥ 95th percentile	0.85 (0.65, 1.12)	0.250	0.89 (0.67, 1.18)	0.430	1.09 (0.39, 3.04)	0.864
MVE (m/s)						
≤ 5th percentile	0.85 (0.65, 1.12)	0.246	0.97 (0.74, 1.28)	0.840	0.33 (0.10, 1.10)	0.071
≥ 95th percentile	1.05 (0.82, 1.34)	0.719	1.06 (0.79, 1.42)	0.702	0.95 (0.61, 1.47)	0.802
LVEF (%)						
≤ 5th percentile	1.00 (0.80, 1.26)	0.978	1.03 (0.81, 1.32)	0.792	0.97 (0.57, 1.68)	0.924
≥ 95th percentile	0.95 (0.74, 1.22)	0.668	1.01 (0.75, 1.34)	0.974	0.80 (0.49, 1.33)	0.396

a Adjusted for maternal age, education, pre-pregnancy body mass index, parity, mode of delivery, HbA1c, gestational age at delivery, birth weight, birth length, infant age, and sex.

b Adjusted for maternal age, education, pre-pregnancy body mass index, parity, mode of delivery, HbA1c, gestational age at delivery, birth weight, birth length, and infant sex.

Extreme values were defined as ≤ 5th or ≥ 95th percentile of the distribution in the control group (infants born to mothers without GDM).

**Figure 3:**
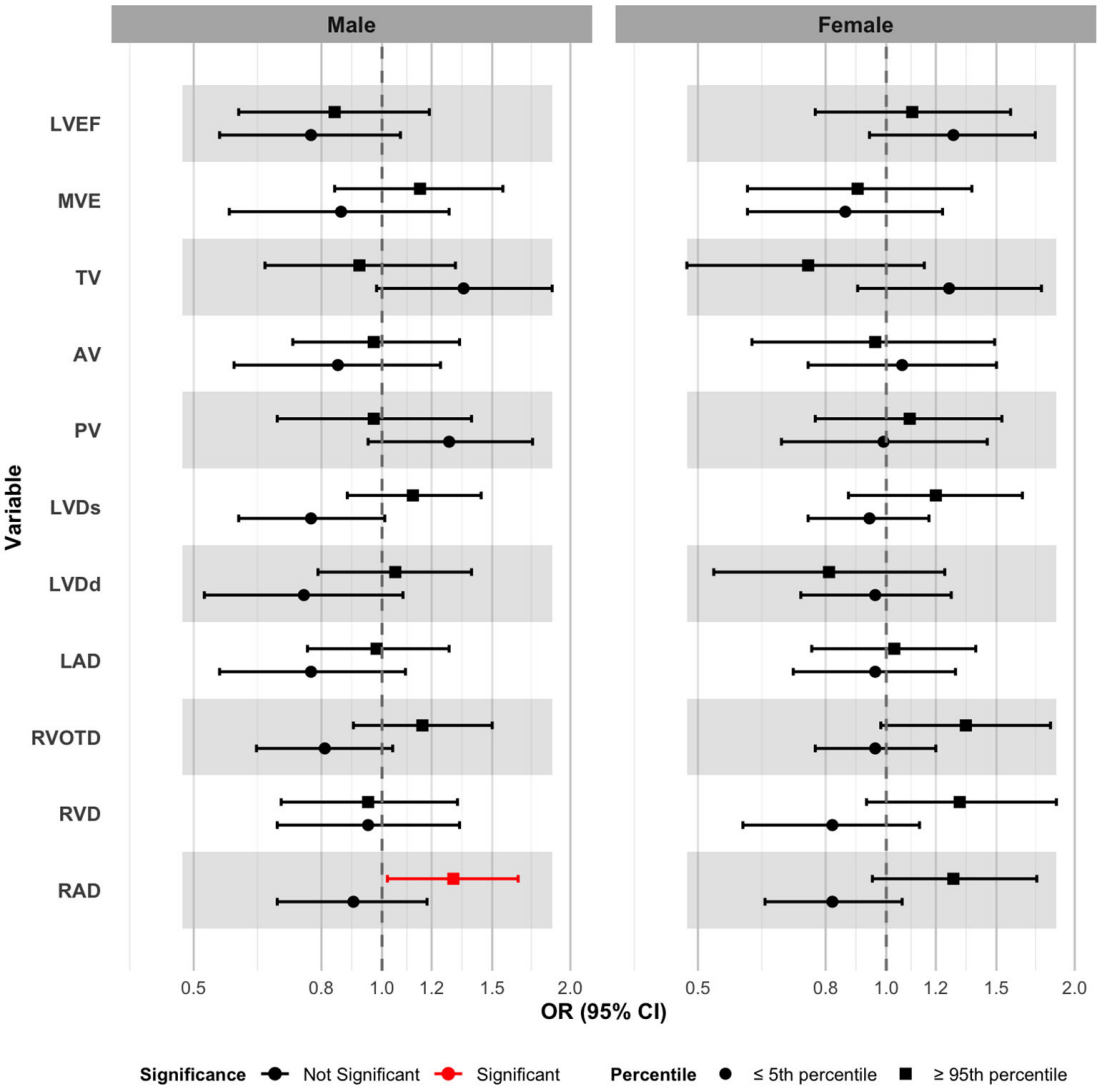
Sex-stratified analysis of odds ratios for extreme values of cardiac structure and function parameters (values ≤ 5th or ≥ 95th percentile) comparing infants born to mothers with GDM with those born to mothers without GDM.

Sex-stratified analysis found that among male infants, GDM exposure was associated with a significantly increased odds of having a RAD ≥ 95th percentile (OR = 1.30, 95% CI: 1.02–1.65, *p* = 0.034), whereas no such association was observed in female infants (**Figure 3**; **Supplementary Table 3**). No statistically significant differences in the odds of extreme values for the remaining cardiac structural and functional parameters were found between sexes. No significant between-group differences were found for the odds of extreme values in LVEF, AV, or PV flow velocities in any of the analyses.

## Discussion

The primary finding of this study is that GDM exposure is significantly associated with alterations in infant cardiac structure, demonstrating notable age and sex specificity. In the overall cohort, GDM was independently associated with increased RAD, RVOTD, LAD, LVDd, and LVDs, as well as with a decreased TV flow velocity. Age-stratified analysis revealed that among infants younger than 6 months, the predominant effects were enlarged cardiac structures and reduced TV flow velocity. In contrast, infants aged 6–12 months exhibited an opposite trend, with smaller RVD and LAD, and an increased TV flow velocity. Stratification by sex indicated that male infants were more susceptible to GDM-related cardiac enlargement, with a significantly increased risk of extremely high values for RAD, whereas no significant associations were observed in female infants. Furthermore, GDM did not have a significant impact on LVEF, nor on AV or PV flow velocities.

The link between poor maternal glycemic control and congenital heart disease in offspring is well-established (19, 20). Furthermore, epidemiological studies indicate that children born to women with GDM have an increased risk of developing early-onset cardiovascular disease during childhood and adolescence (21, 22). Our research and that of others have demonstrated that GDM is associated with morphological and functional changes in the fetal and infant heart, predominantly affecting the right ventricle, consistent with its dominance in late gestation (13, 23, 24). However, it remains unclear whether these cardiac alterations, which arise in response to a relatively mild and transient maternal condition, persist after birth and whether they can identify a subgroup of children at increased long-term cardiovascular risk (25, 26). On one hand, some evidence suggests these effects are transient. Mehta et al., in a study of 50 neonates, found that early cardiac abnormalities, such as myocardial hypertrophy and reduced diastolic function, were temporary and typically resolved within the first month of life (27). On the other hand, some studies have reported more persistent or severe effects. In the study by Aguilera et al., 73 women with GDM and 73 with uncomplicated pregnancies were recruited from the Fetal Medicine Unit of a UK teaching hospital (13). Repeated echocardiograms in their offspring during infancy revealed that GDM was associated with alterations in fetal heart function and structure, including a more globular right ventricle, and that these cardiac changes persisted into infancy (13). Patey et al. observed persistent alterations in left ventricular geometry during the perinatal period in a cohort of 21 neonates (25). Similarly, a retrospective study by Zablah et al. showed that both systolic and diastolic functions of the left ventricle were diminished during the first week of life in 75 neonates exposed to pregestational diabetes or GDM compared with controls (28).

While the absolute differences in cardiac parameters are subtle and do not equate to overt clinical pathology, emerging evidence provides preliminary hints of a potential association between such early subtle structural alterations and long-term cardiovascular risk. As highlighted in a systematic review by Skovsgaard et al., subtle cardiac structural and functional changes in infants exposed to maternal diabetes are associated with an increased risk of left ventricular hypertrophy and hypertension in adolescence, although causal relationships have not been definitively established (15). Similarly, Aguilera et al. reported that GDM-induced fetal cardiac adaptations, which are often subclinical in infancy, may persist as a substrate for long-term cardiovascular vulnerability (13). These studies collectively suggest that subtle early-life cardiac changes could serve as potential precursors of later cardiovascular outcomes, but their clinical relevance in the context of routine pediatric care—particularly in asymptomatic infants—remains to be fully elucidated.

Our study’s age-stratified analysis revealed opposing directions of association between GDM exposure and cardiac parameters in different age subgroups. This complex pattern may reflect a dynamic process of postnatal cardiac remodeling. The initial cardiac enlargement in the younger group is consistent with the established effects of the *in utero* hyperglycemic–hyperinsulinemic state, a core tenet of the fetal programming hypothesis (29, 30). However, the subsequent finding of smaller dimensions in older infants could suggest a “catch-down” growth phenomenon or an adaptive remodeling phase once the infant is removed from the diabetic intrauterine environment (31). Alternatively, this observation could be influenced by other factors not fully captured in our study, such as the timing of measurement relative to physiological changes or variations in postnatal nutrition. A central finding of our study is the sex-specific effect of GDM on infant cardiac structure. Male infants appeared more susceptible to GDM-related cardiac alterations, whereas such associations were not significant in female infants. This observation aligns with sexual dimorphism in developmental programming, although the precise mechanisms driving this male vulnerability remain to be elucidated. The established hypothesis for GDM-induced cardiac changes posits that maternal hyperglycemia triggers fetal hyperglycemia and subsequent hyperinsulinemia. This metabolic disturbance promotes excessive glycogen and lipid deposition in cardiomyocytes, leading to myocardial hypertrophy, the severity of which is thought to correlate with the degree of glycemic control during pregnancy (32). Furthermore, experimental evidence suggests that the GDM intrauterine environment may induce fetal hypoxia, which can directly cause cardiomyocyte injury and apoptosis, ultimately impairing ventricular function (33). Although these mechanisms are generally applicable, they do not fully explain the sex-specific pattern observed in our study. We speculate that this male susceptibility may arise from an interplay between the GDM environment and intrinsic sex-based biological differences. One plausible explanation is the sex-specific placental response to metabolic stress. Male placentas have been shown to be less adaptable and mount a more proinflammatory response to maternal insults, potentially exposing the male fetus to a more hostile intrauterine milieu than its female counterpart (34, 35).

The primary strength of this study was its substantial sample size, which enabled detailed stratified analyses. By stratifying by age (< 6 months vs. 6–12 months) and sex, we confirmed the overall association between GDM exposure and alterations in infant cardiac structure and, more importantly, uncovered the complex dynamic evolution and sex specificity underlying this relationship. Nevertheless, this study has several limitations. First, the retrospective, single-center cohort design is limited to observing associations rather than establishing causality and may introduce selection bias. The data were systematically confined to mother–infant pairs who delivered at our hospital, and information on women who received antenatal care in the catchment area but delivered elsewhere could not be ascertained. This could potentially limit the generalizability of our findings. Furthermore, despite statistical adjustment for several known confounders, we were unable to account for key maternal variables (including socioeconomic status, dietary patterns, and supplement use) or important postnatal factors (such as feeding mode, infant growth trajectories, and early illnesses). As these factors are established determinants of both maternal metabolic health and fetal development, their omission may have contributed to residual confounding. While our study provides valuable insights, the findings should be interpreted with caution and require further validation through prospective studies with more comprehensive data collection.

## Conclusions

Maternal GDM is associated with dynamic, sex-specific, and subtle alterations in infant cardiac structure, with male infants showing greater susceptibility. Although current evidence—including our findings—is insufficient to confirm the direct clinical implications of these changes, they reflect potential early markers of cardiovascular vulnerability rather than established clinical abnormalities. These findings underscore the need for enhanced surveillance of GDM offspring, particularly male offspring, and longitudinal studies to determine whether these early changes predict long-term cardiovascular risk.

## Data Availability

The raw data supporting the conclusions of this article will be made available by the authors, without undue reservation.

## Supplementary material

Supplementary Table 1 Adjusted mean differences comparing cardiac structure and function parameters in infants exposed to maternal GDM with those in infants not exposed to maternal GDM.

Supplementary Table 2 Stratified analysis by sex of adjusted mean differences in cardiac structure and function parameters in infants exposed to maternal GDM compared to those not exposed to maternal GDM.

Supplementary Table 3 Sex-stratified analysis of odds ratios for extreme values of cardiac structure and function parameters (values ≤ 5th percentile or ≥ 95th percentile) comparing infants born to mothers with GDM to those born to mothers without GDM.
