# Supplementary material for: The impact of maternal gestational diabetes mellitus on cardiac structural and functional parameters in infants
Source: Front Endocrinol (Lausanne). 2026 Mar 5;17:1701975. doi: 10.3389/fendo.2026.1701975 (PMC12999395; doi:10.3389/fendo.2026.1701975)
Supplement: Supplementary Table 1 — Adjusted mean differences comparing cardiac structure and function parameters in infants exposed to maternal GDM with those in infants not exposed to maternal GDM. [file Table1.docx]

**Supplementary Table S1** Adjusted mean differences comparing cardiac structure and function parameters in infants exposed to maternal GDM with those in infants not exposed to maternal GDM.

| Variable | All participants | | |  | Aged less than 6 months | | |  | Aged 6-12 months | | |
| --- | --- | --- | --- | --- | --- | --- | --- | --- | --- | --- | --- |
|  | Adjusted mean difference  (95% CI)^*^ | P-value | FDR q-value |  | Adjusted mean difference  (95% CI)^**^ | P-value | FDR q-value |  | Adjusted mean difference  (95% CI)^**^ | P-value | FDR q-value |
| RAD | 0.22 (0.09, 0.36) | 0.001 | 0.011 |  | 0.13 (0.01, 0.26) | 0.04 | 0.11 |  | -0.18 (-0.44, 0.09) | 0.187 | 0.309 |
| RVD | 0.08 (-0.07, 0.23) | 0.318 | 0.398 |  | 0.07 (-0.08, 0.22) | 0.363 | 0.427 |  | -0.37 (-0.72, -0.01) | 0.043 | 0.119 |
| RVOTD | 0.19 (0.08, 0.3) | 0.001 | 0.011 |  | 0.1 (-0.02, 0.22) | 0.09 | 0.165 |  | -0.01 (-0.23, 0.21) | 0.914 | 0.976 |
| LAD | 0.17 (0.03, 0.32) | 0.02 | 0.073 |  | 0.19 (0.04, 0.33) | 0.013 | 0.048 |  | -0.48 (-0.79, -0.17) | 0.002 | 0.011 |
| LVDd | 0.29 (0.11, 0.46) | 0.002 | 0.011 |  | 0.14 (-0.04, 0.32) | 0.121 | 0.199 |  | -0.15 (-0.45, 0.15) | 0.317 | 0.425 |
| LVDs | 0.17 (0.05, 0.29) | 0.006 | 0.022 |  | 0.03 (-0.09, 0.16) | 0.588 | 0.646 |  | 0.07 (-0.15, 0.29) | 0.525 | 0.605 |
| PV | 0 (-0.01, 0.01) | 0.55 | 0.605 |  | -0.01 (-0.02, 0) | 0.249 | 0.332 |  | 0.01 (-0.01, 0.03) | 0.267 | 0.368 |
| AV | 0 (-0.01, 0.01) | 0.623 | 0.664 |  | 0 (-0.01, 0.01) | 0.902 | 0.902 |  | 0 (-0.01, 0.02) | 0.548 | 0.644 |
| TV | -0.01 (-0.02, 0) | 0.019 | 0.073 |  | -0.01 (-0.02, 0) | 0.005 | 0.028 |  | 0.02 (0, 0.03) | 0.027 | 0.083 |
| MVE | 0 (-0.01, 0.01) | 0.593 | 0.644 |  | 0 (-0.01, 0.01) | 0.524 | 0.605 |  | -0.01 (-0.03, 0) | 0.07 | 0.14 |
| LVEF | 0.01 (-0.31, 0.32) | 0.976 | 0.976 |  | 0.15 (-0.22, 0.52) | 0.422 | 0.515 |  | -0.62 (-1.24, 0) | 0.051 | 0.14 |

^*^Adjusted for maternal age, education, pre-pregnancy body mass index, parity, mode of delivery, HbA1c, gestational age at delivery, birth weight, birth length, infant age and sex.

^**^Adjusted for maternal age, education, pre-pregnancy body mass index, parity, mode of delivery, HbA1c, gestational age at delivery, birth weight, birth length, infant sex.

**Supplementary Table S2** Stratified analysis by sex of adjusted mean differences in cardiac structure and function parameters in infants exposed to maternal GDM compared to those not exposed to maternal GDM.

| Variable | Male | | |  | Female | | |
| --- | --- | --- | --- | --- | --- | --- | --- |
|  | Adjusted mean difference (95% CI) | P-value | FDR q-value |  | Adjusted mean difference (95% CI) | P-value | FDR q-value |
| RAD | 0.27 (0.09, 0.46) | 0.004 | 0.018 |  | 0.16 (-0.03, 0.35) | 0.102 | 0.224 |
| RVD | 0.11 (-0.1, 0.31) | 0.315 | 0.385 |  | 0.04 (-0.17, 0.26) | 0.698 | 0.768 |
| RVOTD | 0.24 (0.08, 0.39) | 0.003 | 0.018 |  | 0.13 (-0.03, 0.29) | 0.119 | 0.238 |
| LAD | 0.25 (0.05, 0.45) | 0.015 | 0.033 |  | 0.08 (-0.13, 0.3) | 0.438 | 0.539 |
| LVDd | 0.37 (0.13, 0.61) | 0.003 | 0.018 |  | 0.19 (-0.08, 0.45) | 0.161 | 0.295 |
| LVDs | 0.23 (0.06, 0.4) | 0.007 | 0.026 |  | 0.1 (-0.07, 0.27) | 0.267 | 0.392 |
| PV | -0.01 (-0.02, 0.01) | 0.445 | 0.539 |  | 0 (-0.02, 0.01) | 0.925 | 0.957 |
| AV | 0.01 (0, 0.02) | 0.172 | 0.252 |  | -0.01 (-0.02, 0.01) | 0.408 | 0.539 |
| TV | -0.01 (-0.02, 0) | 0.122 | 0.201 |  | -0.01 (-0.02, 0) | 0.077 | 0.201 |
| MVE | 0 (-0.01, 0.01) | 0.867 | 0.907 |  | -0.01 (-0.02, 0.01) | 0.339 | 0.466 |
| LVEF | -0.01 (-0.45, 0.42) | 0.957 | 0.957 |  | 0.03 (-0.44, 0.5) | 0.907 | 0.957 |

Adjusted for maternal age, education, pre-pregnancy body mass index, parity, mode of delivery, HbA1c, gestational age at delivery, birth weight, birth length, and infant age.

**Supplementary Table S3** Sex-stratified analysis of odds ratios for extreme values of cardiac structure and function parameters (values ≤ 5th percentile or ≥ 95th percentile) comparing infants born to mothers with GDM to those born to mothers without GDM.

| Variable |  | Male | |  | Female | |
| --- | --- | --- | --- | --- | --- | --- |
|  |  | ORs (95% CI) | P-value |  | ORs (95% CI) | P-value |
| RAD |  |  |  |  |  |  |
| ≤ 5th percentile |  | 0.9 (0.68, 1.18) | 0.436 |  | 0.82 (0.64, 1.06) | 0.13 |
| ≥ 95th percentile |  | 1.3 (1.02, 1.65) | 0.034 |  | 1.28 (0.95, 1.74) | 0.103 |
| RVD |  |  |  |  |  |  |
| ≤ 5th percentile |  | 0.95 (0.68, 1.33) | 0.774 |  | 0.82 (0.59, 1.13) | 0.223 |
| ≥ 95th percentile |  | 0.95 (0.69, 1.32) | 0.774 |  | 1.31 (0.93, 1.87) | 0.126 |
| RVOTD |  |  |  |  |  |  |
| ≤ 5th percentile |  | 0.81 (0.63, 1.04) | 0.095 |  | 0.96 (0.77, 1.2) | 0.699 |
| ≥ 95th percentile |  | 1.16 (0.9, 1.5) | 0.259 |  | 1.34 (0.98, 1.83) | 0.066 |
| LAD |  |  |  |  |  |  |
| ≤ 5th percentile |  | 0.77 (0.55, 1.09) | 0.136 |  | 0.96 (0.71, 1.29) | 0.764 |
| ≥ 95th percentile |  | 0.98 (0.76, 1.28) | 0.904 |  | 1.03 (0.76, 1.39) | 0.839 |
| LVDd |  |  |  |  |  |  |
| ≤ 5th percentile |  | 0.75 (0.52, 1.08) | 0.123 |  | 0.96 (0.73, 1.27) | 0.78 |
| ≥ 95th percentile |  | 1.05 (0.79, 1.39) | 0.742 |  | 0.81 (0.53, 1.24) | 0.338 |
| LVDs |  |  |  |  |  |  |
| ≤ 5th percentile |  | 0.77 (0.59, 1.01) | 0.059 |  | 0.94 (0.75, 1.17) | 0.552 |
| ≥ 95th percentile |  | 1.12 (0.88, 1.44) | 0.369 |  | 1.2 (0.87, 1.65) | 0.276 |
| PV |  |  |  |  |  |  |
| ≤ 5th percentile |  | 1.28 (0.95, 1.74) | 0.108 |  | 0.99 (0.68, 1.45) | 0.973 |
| ≥ 95th percentile |  | 0.97 (0.68, 1.39) | 0.878 |  | 1.09 (0.77, 1.53) | 0.624 |
| AV |  |  |  |  |  |  |
| ≤ 5th percentile |  | 0.85 (0.58, 1.24) | 0.393 |  | 1.06 (0.75, 1.5) | 0.728 |
| ≥ 95th percentile |  | 0.97 (0.72, 1.33) | 0.868 |  | 0.96 (0.61, 1.49) | 0.838 |
| TV |  |  |  |  |  |  |
| ≤ 5th percentile |  | 1.35 (0.98, 1.87) | 0.068 |  | 1.26 (0.9, 1.77) | 0.184 |
| ≥ 95th percentile |  | 0.92 (0.65, 1.31) | 0.65 |  | 0.75 (0.48, 1.15) | 0.184 |
| MVE |  |  |  |  |  |  |
| ≤ 5th percentile |  | 0.86 (0.57, 1.28) | 0.448 |  | 0.86 (0.6, 1.23) | 0.399 |
| ≥ 95th percentile |  | 1.15 (0.84, 1.56) | 0.381 |  | 0.9 (0.6, 1.37) | 0.633 |
| LVEF |  |  |  |  |  |  |
| ≤ 5th percentile |  | 0.77 (0.55, 1.07) | 0.124 |  | 1.28 (0.94, 1.73) | 0.114 |
| ≥ 95th percentile |  | 0.84 (0.59, 1.19) | 0.316 |  | 1.1 (0.77, 1.58) | 0.601 |

Adjusted for maternal age, education, pre-pregnancy body mass index, parity, mode of delivery, HbA1c, gestational age at delivery, birth weight, birth length, and infant age.

Extreme values were defined as ≤5th or ≥95th percentile of the distribution in the control group (infants born to mothers without GDM).
