# Supplementary figures and images for: The impact of maternal gestational diabetes mellitus on cardiac structural and functional parameters in infants
Source: Front Endocrinol (Lausanne). 2026 Mar 5;17:1701975. doi: 10.3389/fendo.2026.1701975 (PMC12999395; doi:10.3389/fendo.2026.1701975)

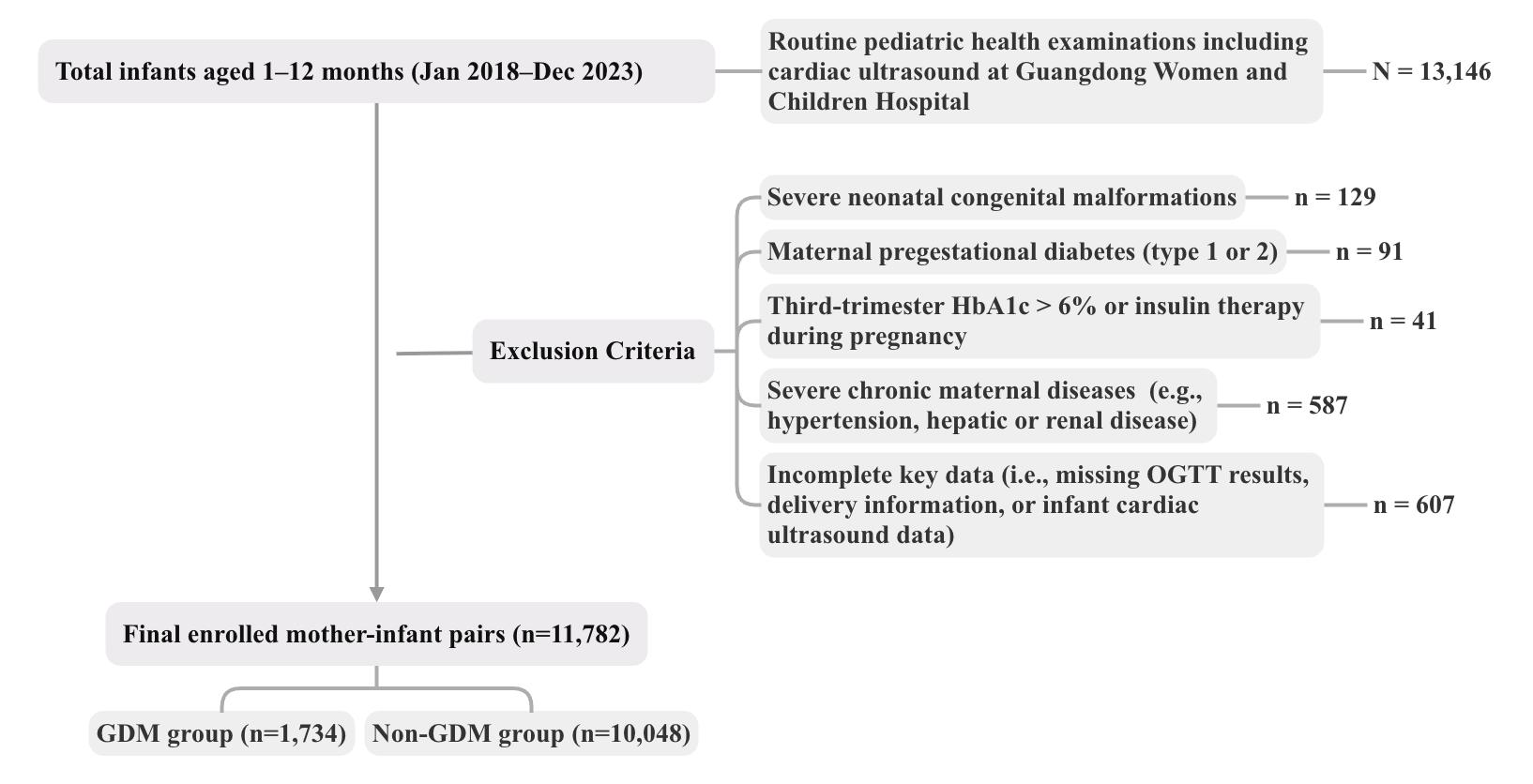

Supplement: Supplementary file 2 [file Image1.png]
